# The LLNA: A Brief Review of Recent Advances and Limitations

**DOI:** 10.1155/2011/424203

**Published:** 2011-06-16

**Authors:** Stacey E. Anderson, Paul D. Siegel, B. J. Meade

**Affiliations:** Health Effects Laboratory Division, National Institute for Occupational Safety and Health, 1095 Willowdale Drive, Morgantown, WV 26505, USA

## Abstract

Allergic contact dermatitis is the second most commonly reported occupational illness, accounting for 10% to 15% of all occupational diseases. This highlights the importance of developing rapid and sensitive methods for hazard identification of chemical sensitizers. The murine local lymph node assay (LLNA) was developed and validated for the identification of low molecular weight sensitizing chemicals. It provides several benefits over other tests for sensitization because it provides a quantitative endpoint, dose-responsive data, and allows for prediction of potency. However, there are also several concerns with this assay including: levels of false positive responses, variability due to vehicle, and predictivity. This report serves as a concise review which briefly summarizes the progress, advances and limitations of the assay over the last decade.

## 1. The Murine Local Lymph Node Assay

Allergic disease continues to be an important environmental and occupational health concern. Allergic contact dermatitis (ACD) is the second most commonly reported occupational illness, accounting for 10% to 15% of all occupational diseases. This poses a significant public health burden with combined annual costs of up to $1 billion for medical costs, workers compensation, and lost time from work. This highlights the importance of developing rapid and sensitive methods for hazard identification of chemical sensitizers. Historically, guinea pig tests (GPT; i.e., the guinea pig maximization (GPMT) and the Buehler assay (BA)) were used for this purpose. The human repeated insult patch test (HIRPT) is still used in many countries as a confirmatory test for skin allergens; however, ethical concerns and the existence of reliable alternative testing procedures have largely eliminated the justification for the HIRPT [1]. The murine local lymph node assay (LLNA) was developed in 1989 [2] and continues to undergo refinement as an alternative for the evaluation of sensitizing potential of low molecular weight (LMW) chemicals. The LLNA has been evaluated extensively in the context of both national and international interlaboratory trials. This data has been reviewed and validated in the USA by the Interagency Coordinating Committee on the Validation of Alternative Methods (ICCVAM) [3, 4] and in Europe by the European Center for the Validation of Alternative Methods (ECVAM) [5] resulting in the LLNA becoming the preferred method for assessing skin sensitization potential by various regulatory authorities [6, 7]. In 2002, the LLNA was adopted by the Organization for Economic Co-operation and Development (OECD) as a standalone method (OECD 429). Recently the LLNA has been designated as the initial requirement for sensitization testing with the new registration, evaluation, authorization and restriction of chemical substances (REACH) regulation in the European Union. 

The basic principle underlying the traditional LLNA is that chemical sensitizers induce a primary proliferation (induction phase) of lymphocytes in the lymph nodes draining the site of chemical application which can be quantified using measurement of radiolabeled thymidine incorporation into the lymph node cellular DNA. Low molecular weight (LMW) chemical sensitizers, referred to as haptens (or prohaptens), are themselves too small to be allergenic and must bind to a protein to be allergenic. Three major cells types, keratinocytes, Langerhans cells, and T-lymphocytes, have been identified as central in the induction phase of ACD. The role of keratinocytes in both the induction and elicitation phases has been recently reviewed [8]. Haptens can directly stimulate keratinocytes present in the epidermis of the skin to release inflammatory mediators such as interleukins 1, 6 and 18, granulocyte-macrophage colony-stimulating factor, and tumor necrosis factor-*α*. The chemokine CCL2, which can recruit dendritic cells into the site of inflammation, is also upregulated in keratinocytes following hapten exposure. Langerhans cells (LCs), immature dendritic cells (DCs) present within the epidermis, take up and process haptenated protein within the major histocompatibility complex (MHC II). In the presence of the proper cytokine-signaling milieu, LCs migrate from the skin through the afferent lymphatics to lymph nodes draining the site of contact and become mature DCs during that process. DCs then present the haptenated peptide to responsive T-lymphocytes [9, 10]. Activated T-lymphocytes divide and differentiate into both T-effector and T-memory cells which starts the central phase of sensitization [11], and it is this allergen-driven proliferation response that is quantified in the LLNA. 

In the performance of the LLNA (based on the original guidelines), mice (female CBA/Ca or CBA/J preferred strain; minimum of four per group) are topically exposed to accepted vehicle, increasing concentrations (minimum of three) of LMW chemical, or accepted positive control on the dorsal surface of each ear once a day for three consecutive days. On day 6, mice are injected, intravenously, via the lateral tail vein with ^3^H-thymidine (^3^H-T). Five hours later, following sacrifice of the left and right draining auricular lymph nodes located at the bifurcation of the jugular vein are excised and pooled for each animal. Lymphocyte proliferation, determined by quantification of radioactive (^3^H-T) incorporation in the draining lymph nodes, is evaluated using a liquid scintillation analyzer. A chemical is classified as a sensitizer if at one or more test concentration it induces a three-fold or greater increase in draining lymph node cell proliferation compared with concurrent vehicle-treated control mice and data follows dose-response kinetics. The data generated from the LLNA has been demonstrated to provide a simple means of obtaining an objective, quantitative evaluation of sensitization. From the analysis generated during the review process, the LLNA was determined to be 86% accurate (*N* = 97), 82% specific (*N* = 33), 87% sensitive (*N* = 93) with positive predictivity of 93% (*N* = 87) when compared to GPT [4]. More detailed descriptions of the LLNA are reported elsewhere [4, 12].

## 2. Benefits over Previously Used Assays for Skin Sensitization

There are many advantages to the LLNA in comparison to GPT (OECD test guideline 406, [13]. The LLNA provides a quantitative endpoint, dose-responsive data, allows for prediction of potency (EC3; effective concentration for a SI of 3 in proliferation of lymph node cells) and does not require the use of an adjuvant. GPT, which evaluate the elicitation phase of skin sensitization provide a qualitative endpoint which tends to be highly variable in part due to its subjective nature, and these tests are not typically used for estimations of potency. Evaluation of the sensitization phase as an endpoint results in a reduced time for animals to be on study and eliminates the discomfort associated with the development of inflammation in the elicitation phase of the response. Compared to GPT, the LLNA reduces animal numbers needed, improves animal welfare, and decreases experimental time and costs. This report is not intended as an exhaustive update on the progress of the LLNA, but rather a concise review which will briefly summarize the progress, advances, and limitations of the assay over the last decade.

## 3. The Potential Use of the LLNA in Risk Assessment 

Although the LLNA was originally validated for the purpose of hazard identification, much attention and debate have been recently focused on the potential use of the LLNA in human quantitative risk assessment [14]. Risk assessment is generally viewed as a four-step process: hazard identification, dose-response assessment, exposure assessment, and risk characterization. The LLNA provides information for use in the first two steps. Lymphocyte proliferation has been proven to be related both causally and quantitatively to chemical sensitization. Potency estimation can therefore be made by comparing the concentration of chemicals necessary for the acquisition of sensitization. The EC3, effective chemical concentration required for a SI = 3, can be mathematically derived by linear interpolation of dose-response data [15]. The EC3 value has been shown to be highly reproducible, consistent among laboratories and stable over time [16–18]. While the EC3 value, is not a measure of absolute potency which can be directly extrapolated to humans, it is an objective measure of relative allergenic potency of one potential sensitizer with that of another [19]. In 2003, after extensive laboratory investigations, four categorizes of chemical sensitization potency, with 10-fold difference in EC3 value (extreme (<0.1%), strong (≥0.1–<1.0%), moderate (≥1.0–<10%), and weak (≥10%)), were determined by the ECETOC (European Center for Ecotoxicology and Toxicology of Chemicals) [20]. Recently, these classification schemes for regulatory purposes have been reevaluated by ICCVAM and the European Chemical Bureau, and their findings will be released later this year [14]. 

The results from numerous studies support the use of the LLNA in quantitative risk assessment by demonstrating an overall association between EC3 and relative potency of chemical allergens in humans [21–25]. EC3 values of 26 chemicals were found to have a linear relationship with the threshold for the induction of sensitization derived from human repeated insult patch test [21]. From the analysis generated during its review process, the accuracy of the LLNA versus human tests (human maximization test and human patch test allergen) was 72% (*N* = 74) while the positive predictivity was 96% (*N* = 51) [4].

There are concerns about extrapolating data generated from the LLNA for potential use in risk assessment. The LLNA is based on the induction phase of the hypersensitivity response following acute (3 consecutive days) exposure. The complexity of the induction-elicitation response and the degree to which skin sensitization influences the dose of chemical necessary to elicit a reaction are important factors to consider. Theoretically, elicitation thresholds are lower than those required for induction. Furthermore, the dose required for induction may be dependent on duration frequency and site of exposure. Human chemical exposure may be the result of an incidental single contact, repeated exposure, or continual exposure. These types of scenarios can present difficulties when trying to classify weak (high EC3) versus strong (low EC3) sensitizers. In general, chemicals with high EC3 are considered to be of low risk to human while chemicals with a low EC3 value present a much higher risk. While this scenario usually holds true, there are other factors that need to be considered such as: is there a greater risk for allergy when there is frequent exposure to a weak sensitizer versus infrequent exposure to a strong or extreme sensitizer? For example, studies have found that although methyl methacrylate is a weak sensitizer (EC3 value of 60–90%), numerous cases of skin sensitization have been reported in individuals exposed to plastic materials [26]. This finding could be related to factors such as duration of exposure, exposure concentration, and route of exposure. In occupational settings, workers have a greater potential for exposure to pure, undiluted chemicals than the general public which would most likely be exposed to a diluted version in a consumer product. While attempts are being made to use data generated from LLNA studies toward utilization in risk assessment, all of these factors need to be carefully considered.

## 4. Limitations of the LLNA 

From the analysis generated during its review process, the accuracy of the LLNA versus GPMT/BA was 89% (*N* = 97), LLNA versus all GPT was 86% (*N* = 126), the LLNA versus human data was 72% (*N* = 74), GPMT/BA versus human was 72% (*N* = 57), and all guinea pig tests (GPT) versus human's was 73% (*N* = 62) [4]. In terms of accuracy, sensitivity, specificity, and positive and negative predictivity, the performance of the LLNA was found to be similar to that of the GPMT/BA. Equally important, the performance of the LLNA and the GPMT/BA was similar when each assay was compared to human data. No predictive toxicology tests will ever be 100% accurate, and because of this, it is important to understand the limitations of each assay [27]. Inconsistencies between LLNA and human patch test data have been documented [28]. While the mouse has been identified as the optimal experimental model for the LLNA, rodents have been shown to have increased skin penetration of chemicals compared to humans [29, 30]. These types of interspecies differences may contribute to some of the inconsistencies between animal and human skin sensitization tests, therefore, confounding interpretation of the results especially with respect to potency determination. This section will briefly describe some of the limitations that have been identified for the LLNA.

### 4.1. Level of False Positives

Irritants and sensitizers can both induce lymphocyte proliferation. While sensitizers generate antigen-specific lymphocyte proliferation, this response is nonspecific for irritants. The use of ^3^H-T incorporation for measurements of lymphocyte proliferation in the LLNA does not allow for differentiation of the two. For this reason, it has long been debated that the LLNA may give an unacceptable number of false positives when nonsensitizing irritating chemicals are tested [17, 31]. The determination of an SI value of 3 as indicative of skin sensitization potential was made after extensive evaluations of chemical datasets. It is a threshold set as a precautionary measure to try and account for background fluctuations in lymphocyte proliferation [15]. For example, topical application of the well-studied surfactant sodium lauryl sulfate (SLS) has been shown to test positive in the LLNA with SI values above the threshold limit (3-fold increase) [9, 17, 31–34]. In contrast to the scenario presented for SLS, when numerous nonsensitizing skin irritants were evaluated using the LLNA, the majority tested negative [35]. However, positive responses, occasionally conflicting with data generated from other studies, to other nonsensitizing irritants have been reported and include: chloroform/methanol, Triton X-100, oxalic acid, methyl salicylate, and nonanoic acid [34, 36]. Similar to SLS, the SI values obtained for these compounds in the LLNA were most often low and close to the threshold level. More recently, 7/9 nonsensitizing irritating compounds (oleic acid, linoleic acid, linolenic acid, undecylenic acid, maleic acid, squalene, and octinol) tested positive in the LLNA with the highest SI value of each substance between the range of 4.4–16.1 [37]. It is important to point out that these types of limitations are not unique to the LLNA and have also been associated with GPT for skin sensitization [38] as well as with human patch test studies [39]. 

Numerous methods have been developed based on the mechanisms underlying the induction of sensitization to try and distinguish between sensitizing and irritating compounds. These include but are not limited to: measurements of antigen expression on Langerhans cells [40], cytokine production [41–43], DC activation [44], and lymph node cell phenotyping [45–48]. At this time, these modifications of the standard LLNA are intended for use as research tools and are not validated for the purpose of hazard identification.

### 4.2. Variability due to Vehicle

Lymphocyte proliferation has also been shown to be influenced by several factors including vehicle selection [49]. OECD recommended vehicles include: acetone/olive oil (AOO: 4 : 1 v/v), dimethylformamide, methyl ethyl ketone, propylene glycol, dimethyl sulphoxide, and dimethyl sulfoxide (DMSO). Several of these vehicles including AOO, DMSO, and propylene glycol have been shown to augment the LLNA response of certain chemicals. For example, AOO has been shown to give highly variable results when used as a vehicle in the LLNA [50, 51]. In addition, research suggests that olive oil itself may cause contact allergy [52, 53]. DMSO is a polar solvent that is known as a penetration enhancer and may augment bioavailability of the allergen across the stratum cornea. Another commonly used LLNA vehicle propylene glycol has been shown to suppress the proliferative effects of certain chemicals such as 2,4-dinitrochlorobenzene (DNCB) [54]. Select vehicles with the ability to enhance or suppress proliferative responses may be an important consideration for weak sensitizers with high EC3 values. Jowsey et al. [55] reported that solvent selection was very important when conducting the LLNA. They tested 15 different solvents with multiple allergens and found that when propylene glycol was used as the vehicle, the EC3 obtained for the chemicals varied by >10 fold compared to the other vehicles used. This is consistent with our study of bromoalkanes [56]. An approximate 3-fold difference in lymph node cell stimulation for C18 and C19 bromoalkanes was observed when dissolved in AOO versus butanol/tetrahydrofuran (1/1). In addition, the importance of other physical/chemical considerations for solvent selection and the potential influences on test results were noted. While allergens may be soluble in AOO, the acetone quickly volatilizes away during application of allergen to the skin. Test compounds such as bromohexane may be lost due to volatility, while the longer chain bromoalkanes result in large particulate-olive oil slurries that are poorly retained on the skin with application in AOO. However, while these are important factors to considerer, the degree of augmentation due to vehicle selection has not typically been shown to affect the category of sensitization because the SI value is based on increase in lymphocyte proliferation over vehicle control.

### 4.3. Inability to Distinguish Specific Type of Hypersensitivity Response

There are two types of chemical allergy which are of greatest relevance for occupational and consumer exposures: skin sensitization causing allergic contact dermatitis (Th1-type immune response) and sensitization of the respiratory tract associated with allergic rhinitis and asthma (Th2-immune response). In addition to identifying contact sensitizers, it is generally accepted that LMW respiratory sensitizers also test positive in the LLNA because the initial sensitization or induction phase of allergy is similar for both types of allergic responses [57]. Based on this concept, a LMW chemical testing negative in the LLNA can be classified as nonsensitizing for urticarial, contact, and respiratory allergies. However, there are currently no validated methods to distinguish between these two types. Modifications of the LLNA have been developed to try and classify the type of chemical sensitizer. These include but are not limited to methods that evaluate serum IgE levels (representative of a Th2-type immune response) [57], cytokine fingerprinting (analysis of Th1 versus Th2 cytokines) [58–60], the mouse ear swelling test (MEST) [61], and immune cell phenotyping [48, 62].

Although there is mounting evidence that lymphocyte proliferation can be used to identify contact and respiratory allergens, dermal application is the only route of exposure validated for the LLNA. While there is significant evidence suggesting that dermal exposure to sensitizers such as the isocyanates and acid anhydrides can induce respiratory tract sensitization [63–65], there are currently no validated test methods to identify compounds, including high molecular weight protein allergens that cannot pass through the skin. Attempts are currently being made to address these issues and will be discussed in greater detail later in this paper.

## 5. What Is in Store for the Future? 

### 5.1. Updated OECD Guidelines

The OECD Guidelines for the testing of chemicals are periodically reviewed as a result of nominations of new methods highlighting scientific progress, changing regulatory needs, and animal welfare considerations. Many modifications to the OECD guidelines have been recently published [66] and will be briefly described in this section. 

### 5.2. Nonradioactive Alternatives

Two modifications of the LLNA to utilize nonradioactive endpoints have been developed. Advantages of these assays include the elimination of occupational exposure to radioactivity and issues related to radioactive waste. The LLNA: DA and LLNA: BrdU have recently been reviewed, validated, and recommended by an international peer review panel as useful for identifying skin sensitizing and non-sensitizing substances, with certain limitations [66]. These methods are considered to be of equal merit to the standard LLNA and may be employed as an alternative to GPT or the standard LLNA. Positive or negative results no longer require additional confirmation. As with all the validated tests discussed, both positive and negative (solvent) controls must be run in parallel to the test substance. The concept for the LLNA: BrdU method (OECD 429B) is similar to that of the standard LLNA and is based on the incorporation and quantification of BrdU into proliferating cells in the auricular lymph nodes following topical chemical exposure [67]. BrdU incorporation is measured by peroxidase-labeled BrdU-specific antibody. Following addition of a substrate, reaction with the peroxidase produces a colored product that is quantified at a specific absorbance using a microtiter plate reader. A chemical is considered a sensitizer if an SI value ≥1.6 is obtained. 

The second approved nonradioactive method is the LLNA: DA (OECD 442A). The LLNA: DA (developed by Daicel Chemical Industries, Ltd.) uses quantification of adenosine triphosphate (ATP) content (known to correlate with living cell number) measured using bioluminescence as an indicator of increased lymphocyte proliferation [68]. The method utilizes the luciferase enzyme to catalyze the formation of light from ATP and luciferin, which is measured using a luminometer and linearly related to the ATP concentration [66]. A chemical is considered a sensitizer if an SI value ≥1.8 is obtained. Although both of these assays provide quantitative data suitable for dose-response assessment, the results may not be directly compared to the EC3 values obtained for the LLNA. The thresholds to determine sensitization (SI) for these assays are lower than that established for the standard LLNA, and SI values for equivalent doses of allergen also tend to be lower. It has not been established, to our knowledge, if this shift in basal and allergen-induced SIs will provide comparable EC3 determinations to the traditional LLNA. As with any assay, there are limitations to these modifications. Certain chemicals, such as ones that affect ATP levels, have been determined to be inappropriate for use with these types of assay. In addition, ATP is very labile and the assay, as presently validated, requires immediate analysis after sample recovery. 

### 5.3. Reduced LLNA

The reduced LLNA (rLLNA) is a validated and accepted modification to the standard LLNA which was purposed in an effort to reduce experimental animal use for the assessment of skin sensitization potential of chemicals as well as to address the demand for increases in sensitization testing of chemicals required by REACH [66]. This alteration of the original protocol requires only a single, high-concentration test group (the highest dose that does not produce significant irritation) and a positive control group [69, 70]. When used to test a substance for the potential to cause allergic contact dermatitis, the rLLNA uses fewer animals than the LLNA to provide a “yes-no” result. ICCVAM has recommended that the rLLNA be used routinely to determine the allergic contact dermatitis hazard potential of chemicals and products before conducting the multidose LLNA in cases that do not require dose-response information. Since the rLLNA uses only a negative control group and a high-dose group, use of the rLLNA can reduce the number of animals needed for each test by 40% compared to the multidose LLNA. Clear justification and scientific rationale must be provided before utilization of this method because it cannot generate dose-response or potency data that could be used for risk assessment. 

### 5.4. Testing of Formulations

The standard LLNA was not originally evaluated for the testing of formulations. However, ICCVAM recently recommended, due to a nomination by the US Consumer Product Safety Commission, to reevaluate the LLNA applicability domain. This would allow the LLNA to be used to test any chemical or product, including pesticide formulations, metals, substances in aqueous solutions, and other products such as natural complex substances and dyes unless the chemical or product to be tested has properties that may interfere with the ability of the LLNA to detect skin-sensitizing substances [71]. This conclusion was based on the compilation of data from previously described research as well as newly generated data obtained for the purpose of this evaluation. For immunotoxicological evaluation of investigational new drugs, the FDA requires that “when a murine LLNA is conducted to support the safety of clinical trials, the sensitizing potential of the drug substance, clinical excipient, and clinical formulation should be evaluated” [72]. This modification expanded the use and application of the LLNA to test formulations found in occupational settings and consumer products as well as individual chemicals. 

### 5.5. Nonanimal Alternatives to the Standard LLNA

Much focus has been placed on the use of *in vitro, in chemico*, and *in silico* alternatives due to the increasing public and political concerns regarding the use of animals in research. The successful development, evaluation, and validation of these nonanimal alternatives for evaluation of skin sensitization will depend heavily on the precision and accuracy with which they can predict the *in vivo* classification of sensitizing chemical. They must be able to predict the complex interaction of the chemical with all aspects of the immune response. Numerous methods are being developed for this purpose and are based on specific mechanistic steps that occur during skin sensitization including but not limited to: protein/peptide binding and haptenization, activation of keratinocytes and DC, and T-cell proliferation [73].

A number of *in silico* methods currently exist and aim to predict a novel chemical reactivity based on the known *in vivo* reactivity of existing structurally similar chemicals. This kind of theoretical computer modeling is referred to as structural activity relationship (SAR) or quantitative structural activity relationship (QSAR). Derek for Windows and TOPKAT are examples that have been used for several years. This type of predictive tool allows the user to input a chemical structure and obtain a readout of the chemical constructs that could potentially lead to sensitization [74]. Challenges with this type of modeling include the analysis of chemicals requiring metabolic activation. Although these models may be helpful as an initial screen, inconsistent results have been observed when compared to animal models of sensitization [75, 76]. 

Computer and skin models are also being developed to evaluate chemical epidermal bioavailability based on physical/chemical data [77]. A chemical must react with host proteins to produce an altered or haptenated selfprotein before sensitization can occur [78–80]. Additionally, some chemicals termed prohaptens require metabolic or chemical conversion before they can react with a protein. Efforts have also focused on investigating whether the intrinsic sensitizing potential of a chemical can be predicted from its electrophilic reactivity [81]. The direct peptide reactivity assay (DPRA) investigates peptide reactivity kinetics to evaluate sensitization and is currently undergoing validation by ECVAM. It aims to model protein haptenation *in chemico*, by measuring the depletion of two synthetic peptides which are typical reaction targets [82]. 

There are numerous *in vitro* tools being developed for prediction of skin sensitizers. Several are based on critical steps in chemical sensitization including keratinocyte and DC activation [73, 77]. These pivotal points of sensitization result in numerous cellular and molecular processes related to antigen processing and presentation which can be evaluated and measured. These include upregulation of costimulatory molecules (CD83, CD86, CD40, and CD80) and various cytokines (IL-1*β*, IL-8, TNF-*α*, and IL-10) which can be measured by methods such as flow cytometry and quantitative real-time PT-PCR. Models are being developed using LC-like dendritic cells derived from human bone marrow, cord blood, or peripheral blood precursors [83, 84] as well as DC-like cell lines including THP1 [84, 85], U937 [86], KG-1 [87, 88] and MUTZ-3 [89]. Limitations with these types of protocols include: donor-to-donor variability (blood-derived DC-), complexity, expense, and lab-to-lab variability.

Two *in vitro* test methods, myeloid U973 skin sensitization test (MUSST) and human cell line activation test (h-CLAT) have been evaluated and accepted for prevalidation as alternatives for evaluating skin sensitization [77] by ECVAM. Limited success has also been obtained when antigen-proliferative responses by naïve T-lymphocytes have been evaluated *in vitro* following coincubation with chemical sensitizer-treated DCs or LCs [90–92]. These types of assays have been able to identify strong but not weak sensitizers. 

It is anticipated that in the next several years a multiparameter system will be available as a nonanimal alternative for the prediction of skin sensitization. Increased confidence for a method would be based on inclusion of analysis of multiple phases of chemical sensitization. 

### 5.6. Modifications of the LLNA to Identify Respiratory Allergens

Given that positive results can be obtained in the LLNA for contact and respiratory allergens [57], numerous *in vitro* and animal models have been investigated to differentiate these responses. However, none are widely applied or fully accepted, most probably due to the complexity of the system and lack of validation efforts [93, 94]. Respiratory allergens are defined by their ability to provoke a Th2-type immune response. While a negative result in the LLNA typically excludes a LMW chemical as a respiratory sensitizer, there is currently no validated screening for the identification of chemicals or proteins that result in allergic sensitization of the respiratory tract. Numerous methods and endpoints have been purposed for the identification of respiratory sensitizers and will briefly be described here. 

It has long been debated which is the best route of exposure when investigating respiratory sensitizers. Although topical application of select strong respiratory sensitizers has been shown to result in sensitization of the respiratory tract, [63–65] this does not hold true for all LMW chemical sensitizers or high molecular weight protein allergens that cannot pass through the skin. Alternative routes of sensitization including intranasal, intratracheal, oropharyngeal, and intradermal have been examined to try and address this issue [95–97]. However, there are several disadvantages with these routes of exposure including chemical solubility (most are not water-soluble), vehicle selection, species variability, requirements for sophisticated equipment and expense have made this a difficult task [57]. These complexities have prevented a general consensus on the best exposure route for evaluation of respiratory sensitizers.

Several endpoints have been examined to try and distinguish respiratory sensitizers from contact sensitizers and are typically based on differences between Th1-(allergic alveolitis/hypersensitivity pneumonitis) and Th2-immune response (allergic asthma and/or rhinitis) [57]. These include the analysis of total serum IgE levels and cytokine fingerprinting [98–100]. While IgE levels tend to be a hallmark of respiratory sensitization and allergic asthma, they do not always correlate to clinical manifestation of asthma [101–103]. This may be due to antigen specificity or failure of the detection of antigen-specific IgE. Identification of respiratory allergen has also been attempted through cytokine profiling. Respiratory allergens generally induce increases in the Th2 cytokines, IL-4, IL-5, IL-10, and IL-13, while contact allergens have been associated with increases in the Th1 cytokines, INF-*γ* and TNF-*α* [58, 104]. This differentiation is not absolute as some respiratory sensitizers have also been shown to increase INF-*γ* [105]. Other asthma models examine lung function along with histopathology, following dermal sensitization and respiratory challenge, for the evaluation of respiratory sensitization [106, 107]. The above-mentioned factors along with method variability have complicated the development of a standardized assay for the identification of respiratory sensitizers. Similar to the LLNA, limitations with these types of models have also been identified and include false positives associated with exposure to respiratory irritants and difficulties with potency measurements [57].

## 6. Conclusions

In conclusion, it should be restated that no toxicology predictive test is perfect, and each will always require a balance between sensitivity and specificity. The LLNA has been recognized as a gold standard for hazard identification of LMW sensitizer for the last decade, and most of the identified limitations are not unique to the LLNA itself but rather to the use of an animal model. Many modifications to the original LLNA OECD guidelines have been published, and others are currently being developed. Among the biggest challenges ahead are maintaining predictive value while moving from whole animal to *in vitro* systems.
